# Exploring the Nonlinear and Threshold Effects of Travel Distance on the Travel Mode Choice across Different Groups: An Empirical Study of Guiyang, China

**DOI:** 10.3390/ijerph192316045

**Published:** 2022-11-30

**Authors:** Mingwei He, Jianbo Li, Zhuangbin Shi, Yang Liu, Chunyan Shuai, Jie Liu

**Affiliations:** Faculty of Transportation Engineering, Kunming University of Science and Technology, Jingming South Road 727, Kunming 650500, China

**Keywords:** travel distance, nonlinear effect, group heterogeneity, mode choice behavior, random forest

## Abstract

Examining how travel distance is associated with travel mode choice is essential for understanding traveler travel patterns and the potential mechanisms of behavioral changes. Although existing studies have explored the effect of travel distance on travel mode choice, most overlook their non-linear relationship and the heterogeneity between groups. In this study, the correlation between travel distance and travel mode choice is explored by applying the random forest model based on resident travel survey data in Guiyang, China. The results show that travel distance is far more important than other determinants for understanding the mechanism of travel mode choice. Travel distance contributes to 42.28% of explanation power for predicting travel mode choice and even 63.24% for walking. Significant nonlinear associations and threshold effects are found between travel distance and travel mode choice, and such nonlinear associations vary significantly across different socioeconomic groups. Policymakers are recommended to understand the group heterogeneity of travel mode choice behavior and to make targeted interventions for different groups with different travel distances. These results can provide beneficial guidance for optimizing the spatial layout of transportation infrastructure and improving the operational efficiency of low-carbon transportation systems.

## 1. Introduction

Advocating for the common efforts of humans to combat climate change and continuously promoting green urban development is the way to build a sustainable society and promote the global achievement of carbon neutrality. However, with the accelerated urbanization and expansion of urban space, increasing travel distance impels residents to choose motorized modes, especially private cars, for their trips. Car-oriented travel demand has made urban traffic congestion, energy consumption and environmental pollution problems increasingly serious [1]. Promoting low-carbon travel modes such as public transportation, walking and bicycling is recognized as an effective method to reduce carbon emissions. However, as the policymakers and planners lack a clear understanding of the effect of travel distance on mode choice behavior, many transportation policies are car-oriented [2], constraining the development of low-carbon transport.

Many studies have explored the relationship between travel distance and travel mode choice and concluded that travel distance is a significant factor in residents’ activity participation and mode choice [3,4]. For example, in short-distance travel, travel mode choice was found to mainly depend on the travel distance [5]. Residents have an acceptable range of how far they can walk or cycle [6]. Once walking or cycling travel distances exceed the acceptable range for travelers, their mode choice behavior may change. The shift from other modes to cars is largely due to increased travel distance and expanding activity space [5]. In addition, there are significant differences in acceptable travel distance and mode choice behavior among groups of different social classes [7,8].

These findings provide important insights into enriching the complex association between travel distance and mode choice. However, current research still has the following issues that must be solved. First, few researchers paid attention to the nonlinear relationship between travel distance and mode choice. It should be noted that pre-specified or parametric relationships may lead to erroneous statistical inferences and policy implications [9]. Furthermore, although some scholars have used the acceptable travel distance obtained by statistical analysis to define the maximum travel distance using walking and cycling, these macro-analyses cannot reflect the micro-characteristics between travel distance and mode choice and are not enough to support the formulation of targeted policy enlightenment considering behavioral heterogeneity across groups. It may weaken the balance of transport policy implementation overlooking the group differences in the effect of travel distance on travel mode choice, which further worsens the risk of social exclusion [10]. Therefore, in order to reduce residents’ dependence on cars and to refine the planning of sustainable communities and cities, it is crucial to understand the possible nonlinear associations and group differences between travel distance and travel mode choice.

Using the resident travel survey and built environment data from Guiyang, this paper employs a random forest model to explore the nonlinear and threshold effects of travel distance on travel mode and the group differences of such effects. We mainly address the following issues: (1) How important is travel distance to mode choice? (2) How does travel distance affect travel mode nonlinearly? (3) How does the nonlinear and threshold effect of travel distance on travel mode vary across groups?

This paper is organized as follows. In Section 2, we review the literature on travel distance and travel mode choice. The research data and methods of the study are described in Section 3. The modeling results are analyzed in Section 4. The main findings are summarized, and related policies are discussed in Section 5.

## 2. Literature Review

The relationship between travel distance and travel mode choice has long been an interesting research topic for transportation scholars. The early modeling of the relationship between travel distance and travel mode choice was mainly based on discrete choice models, especially the widely used multinomial logit (MNL) model [11]. Researchers mostly tend to use the parameter estimates obtained from regression analysis to describe the association between travel distance and mode choice. Most studies show that travel distance matters for travel mode choice [12,13,14]. A study by Dėdelė et al. [7] revealed that travel distance has the most impact on active travel mode choice. As the travel distance increased, the probability of travelers choosing cars increased and the probability of choosing an active travel mode declined [15,16,17]. People who live near workplaces and public transportation were found to be more likely to choose active travel modes [18,19]. In addition, walking and cycling have become effective alternatives to motorized travel over short distances [20], and e-bikes have the potential to replace cars in relatively long-distance travel, which could potentially make a significant contribution to sustainable transport [21,22]. The structural equation model was also used to explore the relationship between travel distances, land use and mode choice through empirical studies [5,23]. These findings confirm that travel distance has a significant impact on travel mode choice, but the exploration of these studies is still limited because we do not exactly know how travel distance affects mode choice.

To guide the layout planning of transportation infrastructure considering the distance and location of travel mode facilities, scholars have studied acceptable travel distances. The concept of acceptable travel distance was proposed by Seneviratne [24]. Arasan et al. [25] regarded the acceptable travel distance as the maximum travel distance by foot and bicycle modes, beyond which people would choose faster travel modes. Seneviratne [24] and Arasan et al. [25] also proposed to use of the cumulative distance frequency distribution (CDF) method to calculate the acceptable distance and regarded the point with the largest rate of change of the curve slope in the cumulative frequency distribution curve of walking distance as the critical distance for walking. In subsequent studies, scholars enriched the research on acceptable distance based on this method. For example, Kim and Ulfarsson [26] determined that the maximum walking distance of American residents was 2.25 km. Scheiner [5] reported the results of a longitudinal analysis of German Resident Travel Survey data from 1976 to 2002 and found that the main alternative to walking for short-distance trips less than 2 km was the private car, while the main alternative for long-distance travel was public transport. Rahul and Verma [27] used statistical analysis to calculate acceptable travel distances of residents in Bangalore, India. The study found that people only used non-motorized transportation when their commuting distance was within 1 km. A private car is the fastest mode for trips over 1 km [2,28]. When travel distance exceeds 10 km, the residents of Kaunas, Lithuania, were 84% and 95% less likely to bike or walk, respectively [7]. Verma et al. [29] considered acceptable travel distances using the Kolmogorov–Smirnov (K-S) fitting test and the cumulative distribution function of time/distance.

The literature also shows significant differences in acceptable travel distances across gender, age, income and other factors [30,31]. Women generally walk and cycle shorter distances than men, and women are more sensitive to the acceptable distance of walking and cycling [7]. There is a significant difference in acceptable travel distances between multimodal commuters and those who rely solely on car commuting [32]. Older adults tend to choose public transportation and walk for short-distance travel compared to other groups [33]. School commuters have lower acceptable distances for walking and cycling than work commuters [34]. The acceptable distances to walk also decreases with increasing income, and travelers whose families have a car have a lower acceptable distance than those without a car [2]. Most of these findings measure acceptable travel distances of walking or cycling by statistical analysis and further explain the group differences in acceptable travel distance. However, most of these studies and analyses focus on a single travel mode, which cannot explain the nonlinear relationship and probability change trend between travel distance and travel mode choice, and the behavioral explanation for multiple travel mode choices is unclear. Therefore, some researchers have also proposed to use of machine learning models to examine the possible nonlinear associations between variables [35].

In recent years, machine learning models have been proven to be a promising alternative in modeling travel mode choices [36,37]. Through data-driven learning, machine learning models can show complex nonlinear relationships between variables and assess the importance of variables. Hagenauer and Helbich [38] and Chang et al. [39] found that travel distance is the most important variable in mode choice determined by machine learning models. In addition, using machine learning models, the scholars also found that travel distance accounted for the highest proportion of explanatory power for predicting student travel modes and short-distance travel modes for car users [14,40]. Liu et al. [1] also found a nonlinear association between travel distance and active travel modes in shopping or commuting trips in the results of machine learning models and found that travel distance has a relative importance of more than 45% for predicting travel activity patterns. This further reveals that the assumption regarding the linear association between travel distance and mode choice of traditional regression models may be seriously violated. However, these findings only illustrate the importance of travel distance and focus on the analysis of the elasticity of land use change. There are some recent studies that have focused on the impact of the built environment near residences and workplaces on the choice of travel mode, such as cars [41], shared bikes [42], using the Metro [43] and customized buses [44]. These studies have profoundly expounded the influence of the built environment on travel behavior, and some scholars have confirmed that the nonlinear relationship and threshold effect between the built environment and travel behavior are likely heterogeneous by gender, income, age, ethnicity and perhaps other characteristics. These heterogeneity analyses can provide theoretical guidance for more targeted traffic policies [45]. However, these studies have not provided any evidence regarding the nonlinear relationship and group heterogeneity between travel distance and mode choice.

Traditional pre-specified or parametric relationship modeling may lead to limitations for urban planners and policymakers in understanding the impact of travel distance on travel mode choice, which has side effects on developing low-carbon transportation modes and building sustainable cities and communities. As far as we know, few studies have revealed nonlinear associations between travel distance and travel mode choice, and there is a lack of exploration of the possible behavioral heterogeneity of nonlinear associations across groups.

## 3. Data and Methods

### 3.1. Data

There were three phases of data collection: in the first phase, the travel information and socio-demographic data of residents were collected. In the second phase, we designed a program to obtain the travel distance. In the third phase, the built environment data regarding the residential locations in the research sample were obtained.

**Figure 1:**
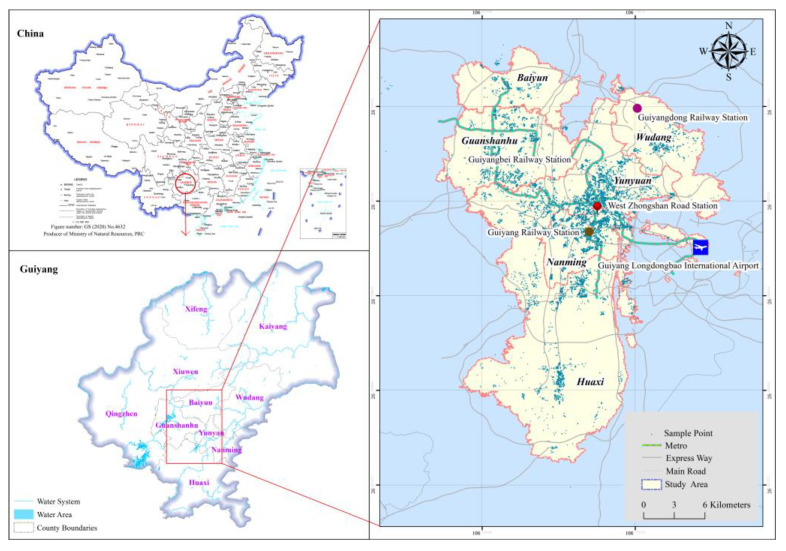
Map of the urban area of Guiyang and the respondents’ household locations.

Travel information and socio-demographic data were collected using a large-scale resident travel survey conducted in Guiyang in January 2021. The survey used the built-in survey program of tablet computers to collect residents’ information. The survey content included three parts: household information, individual characteristics and all personal travel information within 24 h of the previous day. We selected the urban area of Guiyang City as the study area. Figure 1 shows the map of the urban area of Guiyang and the respondents’ household locations. The area of the urban area of Guiyang is approximately 639 km^2^, with a population of roughly 3.17 million, accounting for 52.8% of the total population of Guiyang (about 6 million). In the end, a total of 30,741 trips from 15,169 households in the urban area were used for analysis.

Travel modes included walking, cycling (bicycles and electric bicycles), public transportation (bus, BRT and subway) and car, accounting for 43.12%, 4.89%, 24.08% and 27.91%, respectively. Due to the poor cycling conditions in Guiyang, residents were relatively less likely to choose cycling in their trips compared to the other three modes of transportation. Therefore, we only focused on three major modes: namely, walking (45.34%), public transportation (25.31%) and car (29.36%).

**Figure 2:**
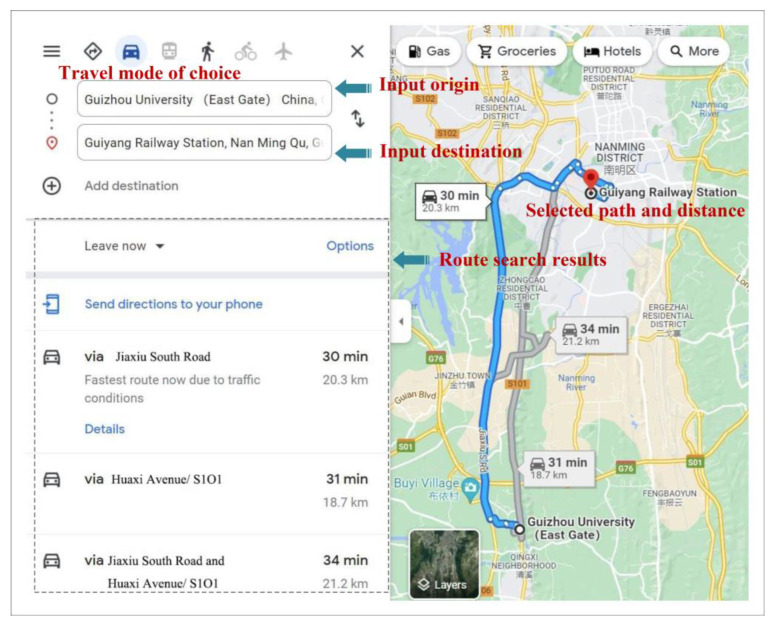
Display of the path search of online map.

Since the ordinary Euclidean distance overlooks tortuous paths and road conditions, calculating the travel distance in this way will lead to serious potential errors. Therefore, we designed a program to obtain more precise travel distances using the “Batch Road Calculation API” function of Baidu Maps. The longitude and latitude coordinates of the residents’ travel origin–destination obtained from the resident travel survey data were input into Baidu Map. The “batch route calculation API” module located the location of the origin and destination through accurate longitude and latitude and output the travel distance of common routes (that is, a route frequently taken by users to meet the needs of most scenarios) in three modes, i.e., walking, public transportation and car (a similar map search case is shown in Figure 2). We regarded the distance of the preferred route as the approximate estimation of travel distance considering the actual residents’ travel modes from origin to destination.

**Figure 3:**
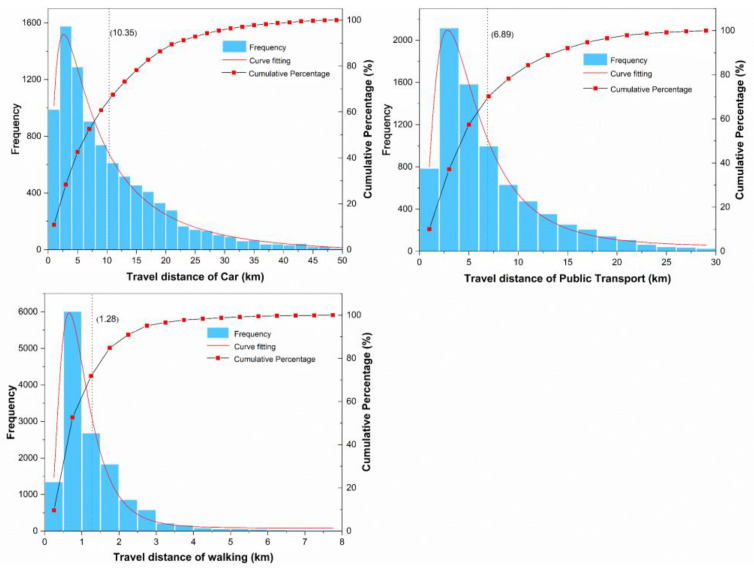
Distance distribution histogram for three travel modes.

According to the estimation results of travel distance based on the “Batch Road Calculation API”, the travel distance distributions of the three travel modes are presented in Figure 3. The travel distance of cars was mainly distributed within 0–25 km, taking up 90% of the total travel distance, with an average of 10.35 km. The travel distance of public transportation was distributed in the range of 0–12 km, taking up 85% of the total travel distance, with an average of 6.89 km. The average walking distance was 1.28 km, and the proportion of walking within 1 km accounted for more than 60%.

The built environment data were collected from Baidu Map, OpenStreetMap, WorldPop and the seventh National Census data. Among them, the population density data were obtained by a combination of both WorldPop (https://www.worldpop.org (accessed on 10 July 2022)) and the seventh National Census data. The road network density data were collected from OpenStreetMap (https://www.openstreetmap.org (accessed on 10 July 2022)). The built environment variables were obtained using ArcGIS software (version 10.6), including land use mix, road network density, population density, distance to CBD and the distance to the nearest bus stop. The road network density and population density were assessed within a 500 m buffer zone. The distance to the nearest bus stop was obtained using the “near” function in ArcGIS software (version 10.6). We adopted the travel distance of the most preferred routes with Batch Route Calculation API as the distance from the house to the CBD. The land use mix is calculated by reclassifying the 17 types of POI data crawled by Baidu Map. We reclassified the 17 types of POIs into five categories: residential, public management and services, commercial services, industry, parks and squares. Based on the 500 m buffer zone, the information entropy, $-\sum_{j}\left(P_{j}ln\left(P_{j}\right)\right)$, was used to indicate the land use mix, where $P_{j}$ is the ratio of the number jth land category to the total number of all POIs. Table 1 presents the explanatory variables for travel mode choice, which include socio-demographics, built environment variables and travel information.

### 3.2. Method

**Figure 4:**
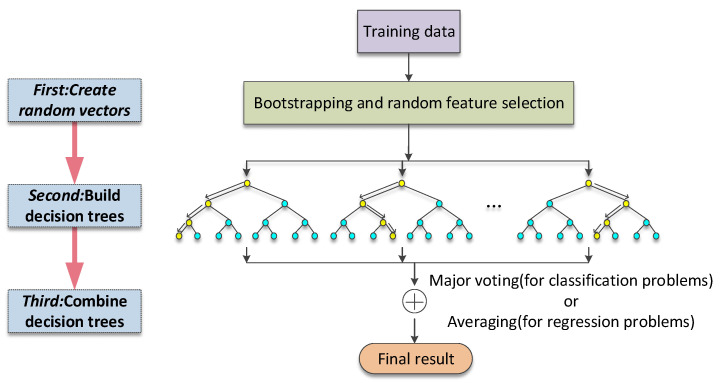
Illustration of random forest algorithm. Source: [35,48].

Random forest (RF) was used in this paper to examine the nonlinear relationship and threshold effects between travel distance and travel mode choice. In recent studies, some scholars have confirmed that random forest has good performance in analyzing travel mode choice. Hagenauer and Helbich [38] compared the prediction performance of seven different learning classifiers and found that random forest (RF) had the highest accuracy. Zhao Xilei [46] provided a comprehensive comparison between machine learning and the logit model in predicting travel mode choice. The results showed that random forest had better prediction performance in dealing with the classification problem of mode choice. This method was proposed by ref. [47], and its basic principle is shown in Figure 4. When performing classification prediction, random forest will generate multiple decision trees. In order to reduce the correlation between decision trees, when the nodes of decision trees are split, random forest will randomly extract the components of training samples and feature vectors. Random forest will hand over the predictor variables to each decision tree for judgment and vote based on the results of each decision tree to decide the final predicting classification. Based on the randomness, the model has good flexibility and generalization ability, which can eliminate the overfitting of the data to a certain extent and reduce the mean square error. Compared with traditional statistical models, although the random forest model cannot generate t-statistics, p-values and other significant indicators [48], this algorithm is more inclusive to the endogenous problems of variables [49]. The algorithm has the advantages of non-parametric characteristics, high accuracy, can handle high-dimensional data and large data sets, and can also evaluate the relative importance of feature variables. In dealing with classification and prediction problems, random forest has been widely used.

In the RF algorithm, the relative importance of explanatory variables is assessed based on the reduction of the Gini index.

Assuming that there are J feature variables, B decision trees and K categories, the Gini index score of each feature $X_{j}$ is represented by $VIM_{j}^{\left(gini\right)}$. Then, the Gini index of the bth tree at node q is calculated by Formula (1).

(1) $$GINI_{q}^{\left(b\right)}=1-\overset{\left|K\right|}{\underset{k=1}{\sum }}P_{qk}^{2},$$

where $P_{qk}$ represents the proportion of category k in node q.

The importance of feature $X_{j}$ at node q of the bth tree is the amount of change in the Gini index before and after the branching of node q is expressed as Formula (2):

(2) $$VIM_{j}^{\left(gini\right)\left(b\right)}=GINI_{q}^{\left(b\right)}-GINI_{l}^{\left(b\right)}-GINI_{r}^{\left(b\right)},$$

where $GINI_{l}^{\left(b\right)}$ and $GINI_{r}^{\left(b\right)}$ represent the Gini indices of the two new nodes after branching, respectively. Finally, the importance obtained for all variables is scored and normalized to obtain the relative importance score of each feature $X_{j}$:

(3) $$VIM_{j}^{\left(gini\right)}=\frac{\sum_{b=1}^{B}\sum_{q\in Q}VIM_{j}^{\left(gini\right)\left(b\right)}}{\sum_{\overset{\prime }{j^{\prime }}=1}^{J}VIM_{j^{\prime }}^{\left(gini\right)}}=\frac{\sum_{b=1}^{B}\sum_{q\in Q}VIM_{j}^{\left(gini\right)\left(b\right)}}{\sum_{\overset{\prime }{j^{\prime }}=1}^{J}\sum_{b=1}^{B}\sum_{q\in Q}VIM_{\overset{\prime }{j^{\prime }}}^{\left(gini\right)\left(b\right)}}.$$

In addition to the relative importance of variables, random forest can characterize the marginal effects of feature variables on dependent variables through partial dependence plots (PDP). Different from traditional statistical models, this relationship does not require prior assumptions and can better reflect the real relationship between independent variables and dependent variables. Assuming that the dependent variable is $Y=f\left(X\right)$, the partial dependence function $\hat{\phi }\left(X_{1}\right)$ of the characteristic variable $X_{1}$ can be expressed as Formula (4):

(4) $$\hat{\phi }\left(X_{1}\right)=\frac{1}{T}\overset{T}{\underset{i=1}{\sum }}f\left(X_{1},X_{i2},\ldots ,X_{ij}\right),$$

where T represents the number of instances in the dataset, and $\left(X_{i2},X_{i3},\ldots ,X_{ij}\right)$ represents the actual observed value of the jth feature of sample i. In this paper, partial dependence plots were used to show the partial effects of travel distance and other variables on mode choice.

## 4. Results

The random forest algorithm was implemented using the “randomForest” toolkit in the R software 4.1.3. We first divided the sample set into a training set and test set according to 7:3. Then, we tested different combinations of the number of split variables (from 2 to 10 with an interval of 1) and the number of decision trees (from 10 to 500 with an interval of 20) to determine the optimal parameters with the smallest out-of-bag error (OOB). The testing results showed that when the number of split variables was 4 and the number of decision trees was 100, the model performed optimally with an out-of-bag error (OOB) of 17.04%. The random forest model established with the above parameters was used for further analysis.

In order to illustrate the impact of the travel distance variable on mode choice and model performance, we used the same training data and test data to establish two random forest models and two multinomial logit (MNL) models. The four models are described as follows:

RF-Mode1: The random forest model considering travel distance variable in travel mode choice modeling.

RF-Mode2: The random forest model without considering travel distance variable in travel mode choice modeling.

MNL-Mode1: The multinomial logit model considering travel distance variable in travel mode choice modeling.

MNL-Mode2: The multinomial logit model without considering travel distance variable in travel mode choice modeling.

### 4.1. Comparison of Model Prediction Performance

In order to compare the performance differences between the random forest model and the multinomial logit (MNL) model in predicting the travel mode choice, we used four evaluation indicators: accuracy, precision, recall and F1 value (F1 score) [50]. The four indicators are calculated as follows:

(5) $$Accuracy=\frac{\sum_{j=1}^{K}\left(TP_{j}+TN_{j}\right)}{\sum_{j=1}^{K}\left(TP_{j}+TN_{j}+FP_{j}+FN_{j}\right)},$$

(6) $$Precision=\frac{\sum_{j=1}^{K}\left(TP_{j}\right)}{\sum_{j=1}^{K}\left(TP_{j}+FP_{j}\right)},$$

(7) $$Recall=\frac{\sum_{j=1}^{K}\left(TP_{j}\right)}{\sum_{j=1}^{K}\left(TP_{j}+FN_{j}\right)},$$

(8) $$F1\;score=\frac{2\times Precision\times Recall}{Precision+Recall},$$

where $TP_{j}$ is the number of jth travel mode correctly predicted as positive samples, $TN_{j}$ is the number of jth travel mode correctly predicted as negative samples, $FP_{j}$ is the number of jth travel mode incorrectly predicted positive samples and $FN_{j}$ is the number of jth travel mode incorrectly predicted negative samples.

The results of the four evaluation indicators in the four models are shown in Table 2. As can be observed, the prediction performance of the random forest model outperformed that of the multinomial logit model regardless of whether the travel distance variable was considered. 

We found that the accuracy, precision, recall and F1 score all significantly decreased after the travel distance variable was not considered in the model. Overall, the random forest model with the travel distance variable (RF-Mode1) had the best prediction performance.

### 4.2. Relative Importance of the Explanatory Variables

**Figure 5:**
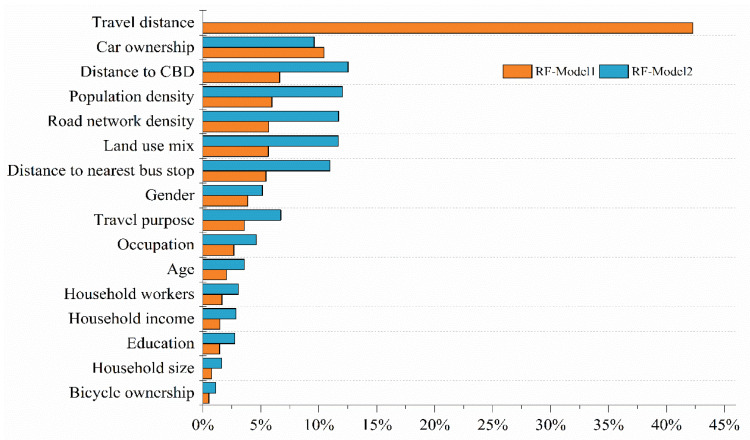
Comparison of relative importance of feature variables.

We present the relative importance of the explanatory variables in the two random forest models (RF-Model1 and RF-Model2) in Table 3 and compare them in the bar chart in Figure 5. We found that in RF-Model2, the relative importance of the built environment was the highest, reaching 58.96%, the relative importance of socio-demographic attributes was 34.32% and the contribution power of travel attributes was only 6.72% in predicting travel mode choice. However, this result did not hold in RF-Model1. We found that the relative importance of the built environment decreased significantly, from 58.96% to 24.80%, when the travel distance variable was taken into account in the model. Travel distance for predicting travel mode choice was more important than built environment variables, with a contribution of 42.28%, similar to other recent studies (e.g., Liu et al. [1]). For the change in the relative importance of the built environment in the two models, a possible reason is that there was an endogenous correlation between the built environment and travel distance. When the travel mode choice model was constructed without considering the travel distance variable, the importance of travel distance for travel mode choice was replaced by the built environment. That is, travel distance may be influenced by the built environment, and the influence of the built environment on travel mode choice may be indirect [5,23]. The relative importance of car ownership also varied significantly. After taking the travel distance variable into the model, the relative importance of car ownership rose from the sixth (9.61%) to the second (10.43%). The reason is not clear, and further research is required to confirm this phenomenon. Among the five variables of the built environment, the largest contribution was the distance to CBD (6.62%), followed by population density (5.96%), road network density (5.68%), land use mix (5.65%) and the distance to the nearest bus stop (5.44%). Among socio-demographic attributes, gender (3.86%) and occupation (2.68%) were also relatively important. The contribution of age (2.03%) to the travel mode choice exceeded that of household income (1.47%) and education (1.43%).

Furthermore, the relative importance of the characteristic variables in RF-Model1 among the three travel modes is shown in Table 4. We found that the importance of these feature variables was not entirely consistent across the three travel modes. Compared with cars and public transportation, the relative importance of travel distance to walking was the highest, reaching 63.24%. This is because with the increase in travel distance, the physical strength consumed by walking will increase, and the distance decay effect will be more obvious [51]. Travel purpose had the second-highest relative contribution in the walking choice (accounting for 5.75%). The importance of car ownership was also different among the three travel modes. Car ownership had the highest contribution in predicting car choice, reaching 29.22%, but its relative importance in predicting walking choice was only 3.44%. In addition to this, the importance of the built environment varied across the three travel modes, which had the highest relative importance for public transportation at 19.69%, while 15.83% and 16.54%, respectively, for cars and walking.

### 4.3. Nonlinear Effects of Travel Distance

**Figure 6:**
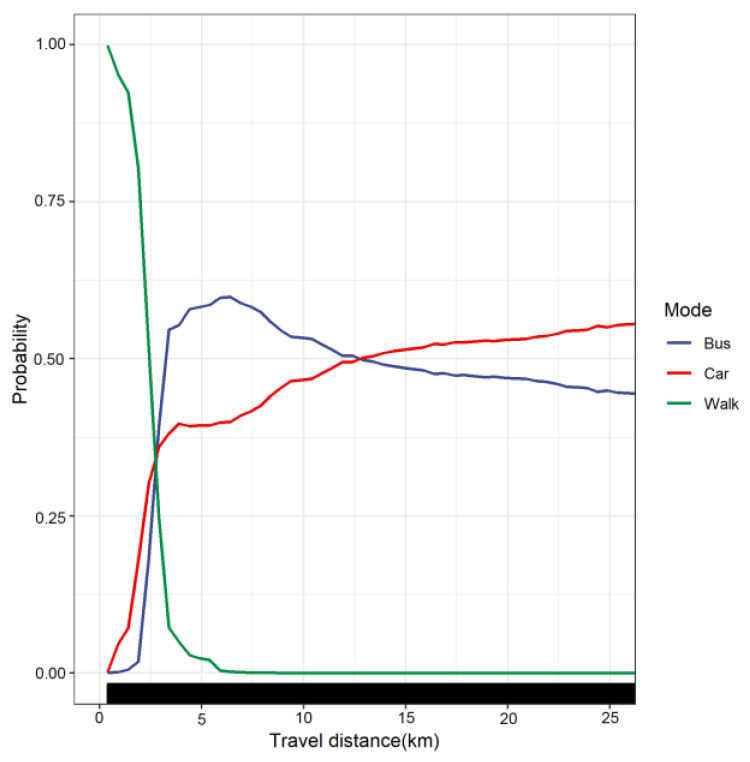
Nonlinear effect of travel distance on travel mode choice.

The nonlinear relationship between travel distance and travel mode choice is illustrated using partial dependence plots (PDP). In Figure 6, the X-axis represents travel distance, and the Y-axis represents the choice probability of travel modes.

Figure 6 provides strong evidence of the nonlinear effect of travel distance on travel mode choice when controlling for other feature variables. Specifically, the choice probability of walking was negatively associated with travel distance. The choice probability of walking within 1.4 km decreased slowly. From 1.4 km to 3.5 km, the choice probability of walking decreased sharply in the form of almost linear change (decreased from 92% to 5%). After that, the walking probability decreased slowly and was below 5% when the travel distance exceeded 4 km. This shows that travelers tend to use walking for short-distance travel. The choice probability of cars increased nonlinearly. At 0–3.5 km, the choice probability of cars increased sharply; at 3.5 km, the proportion of car choice probability reached 38%; beyond 3.5 km, the choice probability increased slowly. There was an obvious threshold effect between the choice probability of public transportation and travel distance. It increased rapidly in the range of 1.25–3.5 km, then increased slowly and reached a peak at approximately 6 km. Additionally, we found that the choice probability of public transportation exceeded 50% in the range of 3.5–6 km. When the distance threshold of 6 km was exceeded, the probability began to decrease gradually. This is inconsistent with previous findings on the positive relationship between travel distance and public transportation choice [7]. In general, we found that before 2.5 km, the choice probability of walking was greater than that of car and public transportation. Especially within 1.4 km, the probability of walking exceeded 90%, and walking took priority in residents’ short-distance trips. Within 2.5–12.5 km, travelers chose public transportation more than cars. Additionally, when the travel distance exceeded 12.5 km, cars were more advantageous than public transportation, with the probability of choosing cars exceeded 50%.

### 4.4. Nonlinear Effects of Built-Environment Variables

In this section, the nonlinear relationship between the built environment and travel mode choice is elaborated. We compared the nonlinear effects of the built environment on travel mode choice in the random forest model with travel distance variables (RF-Model1) and the random forest model without travel distance variables (RF-Model2) by partial dependence plots (PDP). The X-axes represent the explanatory variables, and the Y-axes represent the choice probability of mode. We aimed to compare the nonlinear changes in the built environment in the two models so as to highlight the importance of travel distance in travel mode choice.

**Figure 7:**
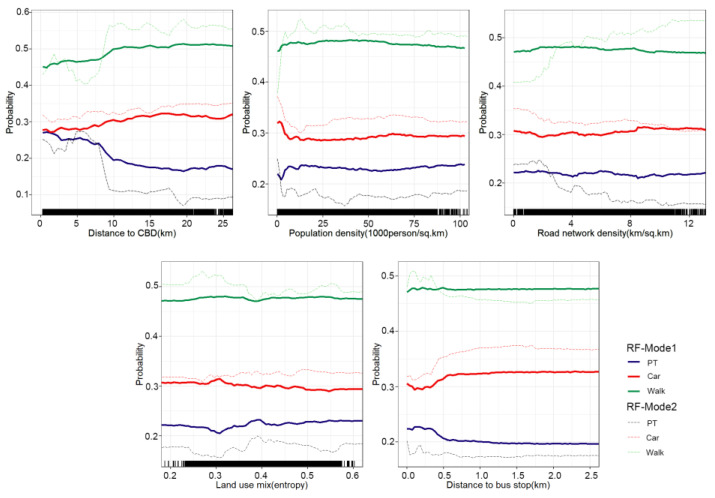
Nonlinear effects of built environment on travel mode choice.

The PDPs of the five built environment variables are shown in Figure 7. Similar to most current studies (e.g., (Ding et al. [52]; Yang et al. [53])), we did not consider the travel distance variable in RF-Model2. We found that the built environment had a significant impact on travel mode choice in RF-Model2. For example, distance to CBD showed a positive correlation with the choice of cars. There were two peak-like nonlinear effects between the distance to CBD and the choice probability of walking. We observed that the choice probability of walking showed an upward trend when the distance from the residence to the CBD was in the range of 0–2 km and 6–10 km. Residents living within 6–10 km of the city center may have been closer to the International Eco-Conference Center Station (the new CBD in Guiyang, approximately 9 km away from the West Zhongshan West Road station), but we still observed that residents closer to the center had a lower probability of walking. Another interesting phenomenon was that with the increase in the distance from CBD, public transport and walking also seemed to show obvious competitive changes. This finding is inconsistent with most existing studies (e.g., (Chen et al. [54]; Eldeeb et al. [55])), but it is reasonable. First, unlike the highly concentrated employment distribution in western countries such as the United States, Guiyang, as a typical mountainous city in Western China, may have more dispersed employment and business distribution. Dispersed and uniform land use may expand the distance between residences and workplaces, causing residents living near CBD to have a low probability of walking. Secondly, as large service complexes, Zhongshan West Road station and the International Eco-Conference Center Station often have a relatively large service range, and the public transport service facilities within the service range are relatively perfect. In areas far away from CBD, due to relatively imperfect public transport service facilities, the probability of residents choosing public transport was low, and the probability of residents’ short-distance walking remained high.

When the population density increased from 0 to 12,500 people/km, the probability of walking increased by approximately 15 percentage points, but the probability of cars and public transportation decreased rapidly. When the population density exceeded 12,500 people/km, the choice probability of cars started to increase, and walking gradually stabilized. This is consistent with the study of Eom and Cho [56]. When road network density increased from 0 to 2 km/km^2^, the choice probability of public transportation increased by approximately 1 percentage point, and walking increased by roughly 2 percentage points, while the choice probability of cars decreased. This is reasonable because lower road network density means less convenience and accessibility by car, and residents tend to choose public transportation and walking [54]. When the land use mix degree increased, the choice probability of cars gradually increased, while public transportation and walking also showed obvious competitive changes. When the land use entropy index was 0.38, the choice probability of walking was the lowest (approximately 47%). After 0.38, the increase in land use mix degree had a weak positive effect on the probability of walking choice [1]. Transit accessibility of house areas can reduce the choice probability of cars and promote the choice of walking within a threshold of approximately 150 m.

However, we found that once the travel distance variable was considered in RF-Model1, the nonlinear effect of the built environment on mode choice seemed to become relatively weak compared with RF-Model2. In particular, the nonlinear effects of land use mix, road network density and distance to the nearest bus stop were not obvious. The nonlinear effect of travel distance on the distance to CBD was also significant. We found that in RF-Model1, the nonlinear effect of distance to CBD on walking and public transport did not show a significant peak change. In addition, among the three travel modes, the nonlinear change of built environment on the probability of walking choice seemed to be most obviously influenced by the variable of travel distance. We found that after considering the variable of travel distance when modeling mode choice, the built environment variables had little nonlinear effects on walking. This finding may again demonstrate the importance of travel distance for predicting travel mode choice, and that travel distance may weaken the non-linear effects of the built environment on travel mode choice.

### 4.5. Group Heterogeneity in Nonlinear Effects of Travel Distance

In Section 4.2, Section 4.3 and Section 4.4, we confirmed that travel distance accounts for the highest proportion when predicting travel mode choice and explored its nonlinear and threshold effects on travel mode choice. Next, we focused on analyzing group differences in the nonlinear effect of travel distance on travel mode choice. We used R software to draw the partial dependence plots (PDP) between travel distance and mode choice for residents in different social groups, including gender, age, occupation, education level, household income, car ownership and travel purpose.

#### 4.5.1. Gender Groups

**Figure 8:**
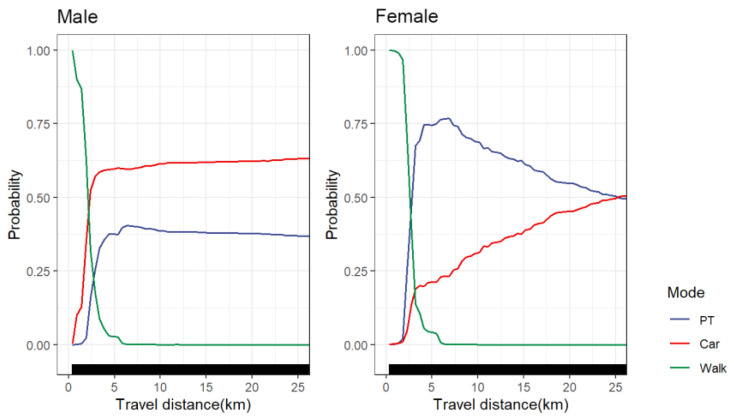
PDP plot of gender groups differences.

Figure 8 shows the nonlinear effect of travel distance on travel mode choice between male and female groups. We found that the nonlinear effect of public transportation and car choice probability was significantly different between males and females. The probability of male groups choosing cars increased sharply at 0–3 km. At 3 km, the proportion of car choice probability reached 60%. After 3 km, the choice probability of cars increased slowly; after 15 km, the choice probability of cars was stable at 62.5%. Consequently, cars seem to be more popular with men [20]. Compared with the male group, the female group rarely chose cars and public transportation within 2 km, mainly using walking. In developing countries, women usually bear more family responsibilities than men. In order to save travel costs, they will be forced to choose employment or activities closer to their residential areas. As a result, walking has become the main mode of transport for short-distance activities. The probability of the male group choosing public transportation peaked at approximately 6 km (about 40%). Once the travel distance exceeded 6 km, the probability of the male group choosing public transportation decreased slowly and stabilized at 37.5% after approximately 15 km. Unlike men, women mainly relied on public transport for long-distance trips. The probability of women choosing public transportation peaked at approximately 7 km (roughly 75%). When the travel distance exceeded 7 km, the probability of women choosing public transportation began to decline sharply. This could suggest that women seem more sensitive to the travel distance of public transportation than men in long-distance trips [57]. A recent study showed that public transportation congestion and lack of safety are the most concerning problems for women using public transportation [58]. This phenomenon needs to be of particular concern to policymakers in order to promote long-distance mobility and traffic equity for women.

#### 4.5.2. Car Ownership Groups

**Figure 9:**
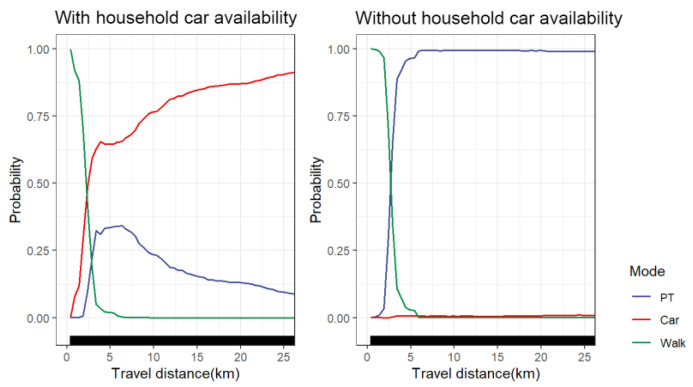
PDP plot of car ownership groups differences.

Figure 9 shows the nonlinear effect of travel distance on travel mode choice between travelers with household car availability and travelers without household car availability. We found that the probability of travelers with household car availability choosing cars increased sharply within the range of 0–4 km. After approximately 2.5 km, the probability of choosing cars exceeded 50%. At 10 km, the probability of travelers with household car availability choosing cars exceeded 75%. It can be seen that in short-distance travel, car use still accounts for a high proportion of travelers with household car availability, and they rely more on cars for trips. Unlike travelers with household car availability, travelers without household car availability rely more on walking within 0–2 km. Additionally, they still rely mainly on public transportation for long-distance travel. We found that the probability of choosing public transportation for travelers without household car availability increased sharply from 0–6 km and approached 100% at 6 km. The probability of choosing public transportation for travelers with household car availability peaked around 6 km, accounting for only 35%. When the travel distance exceeded 6 km, the probability of taking public transportation for travelers with household car availability began to decrease. Influenced by affordability, travelers without household car availability were less likely to own cars and driver’s licenses, and the shift in travel patterns to cars was much weaker [5]. From this perspective, it seems beneficial for the government to reduce the use of cars by limiting household car ownership. However, in order to meet the medium and long-distance trip requirements of car travelers without household car availability, it is important to increase the supply of public transport facilities and implement public transport financial compensation policies for them.

#### 4.5.3. Age Groups

**Figure 10:**
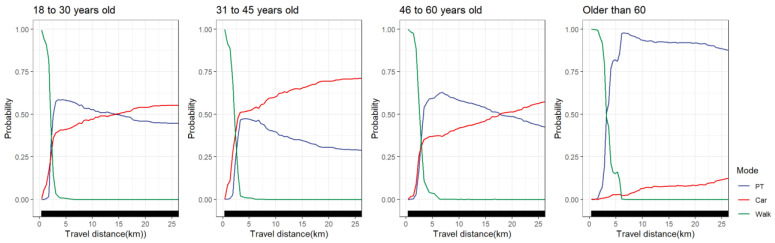
PDP plot of age groups differences.

Figure 10 shows the difference in the nonlinear effect of travel distance on mode choice varying between age groups. It can be seen that the probability of travelers aged 45 and below choosing to walk was almost zero when the travel distance exceeded 3 km. However, at 3 km, 46- to 60-year-old travelers still had a 12.5% probability of choosing walking. Compared with other age groups, the elderly over 60 years old were more likely to choose walking within 3 km, and the downward trend of walking was relatively gentle. Travelers aged 31–45 were more likely to choose cars than other groups, and the probability of choosing cars exceeded 50% at 3 km. However, the probability of the elderly over 60 choosing to travel by car was less than 15%. We found that among travelers aged 18 to 30 and 31 to 45, the choice probabilities of public transportation reached a peak at approximately 3 km. The difference was that the probability of 18- to 30-year-old travelers choosing public transportation at 3 km was more than 60%. However, the probability of 31- to 45-year-old travelers choosing public transportation at 3 km was less than 50%. For travelers aged 46 to 60 and over 60, the choice probability of public transportation showed an upward trend from 0 to 6 km and peaked at around 6 km. The probability of the elderly over 60 years old choosing public transportation at 3 km was 50% and was more than 90% at 6 km. In general, compared with other age groups, the elderly over 60 years old use cars less in trips due to physical limitations and driving age, and they use walking and public transport more, especially in medium and long-distance travel; in conclusion, they rely more on public transportation. Policymakers should pay more attention to the behavioral differences between the elderly and other groups, and targeted planning and layout should be conducive to the walking and public transportation environment for the elderly.

#### 4.5.4. Occupational Groups

**Figure 11:**
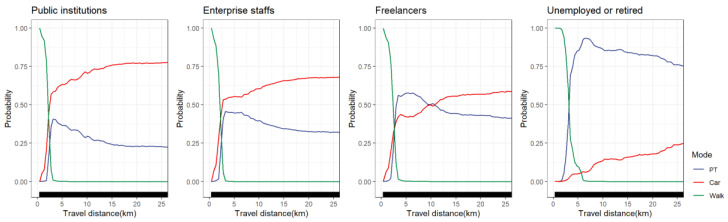
PDP plot of occupational groups differences.

Figure 11 shows the non-linear effects of travel distance on travel mode choice varying across occupational groups. We found that the changing trend of walking choice probability was basically the same among working travelers (including public institutions and civil servants, enterprise staff and freelancers). When travel distances exceeded 3 km, working travelers hardly chose to walk. However, unemployed or retired groups had a 50% probability of walking at 3 km. Among working travelers, cars still accounted for a high proportion within 2.5 km. In particular, among public institutions, civil servants and enterprise staff, the probability of choosing cars was higher than that of public transportation. For freelancers, the probability of choosing public transportation exceeded 50% at 3 km and then slowly increased to a peak at 5 km (approximately 56%). The probability of choosing public transportation began to decline after 5 km. After approximately 10 km, the probability of freelancers choosing public transportation was below 50%, and they tended to choose cars. Different from working travelers, the probability of unemployed or retired travelers choosing cars within the range of 0–25 km was less than 25%. In medium and long-distance travel, unemployed or retired travelers tended to use public transportation and the probability of choosing public transportation peaked at 6 km (accounting for more than 90%). It is understandable that most unemployed or retired travelers belong to the vulnerable class who lack the ability to travel, and they are likely to be limited by affordability and physical conditions. Public transportation is their preferred mode of transportation for medium and long-distance travel.

#### 4.5.5. Educational Level Groups

**Figure 12:**
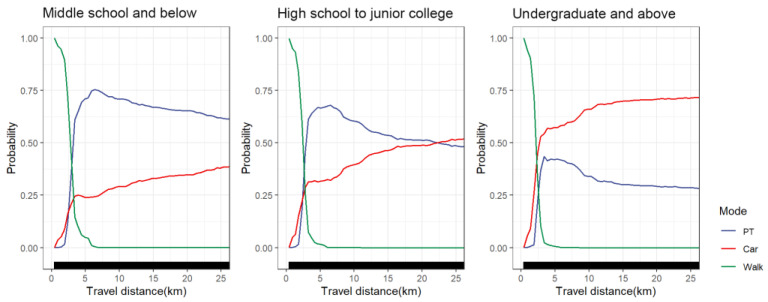
PDP plot of educational level groups differences.

As shown in Figure 12, the nonlinear effect of travel distance on car choice and public transportation choice differs significantly across educational level groups. Specifically, at 0–3 km, the probability of choosing cars showed an upward trend among the three groups. The difference was that the chosen probability of cars at 3 km was more than 50% for travelers with higher education levels (undergraduate and above), while it was 30% for travelers with secondary education levels (high school to junior college) and only 25% for travelers with lower education levels (middle school and below). After 3 km, the probability of choosing cars increased slowly among the three groups. Among travelers with higher education levels, the probability of choosing cars was higher than that of public transportation. Although the probability of travelers with higher education levels choosing public transport peaked at 3 km, it only accounted for 40%, and cars seemed to be more popular among them. Unlike travelers with higher education levels, public transportation still had a great advantage in medium and long-distance travel for low and medium-educated travelers. Public transportation choice probability peaked at approximately 5 km (about 65%) for travelers with high school to junior college diplomas. The public transportation choice probability peaked at approximately 6 km (around 75%) for travelers with middle school diplomas and below. For low-education travelers, who may have lower income levels, the unaffordable cost of car trips forced them to choose public transportation more often for medium and long-distance trips.

#### 4.5.6. Household Income Groups

**Figure 13:**
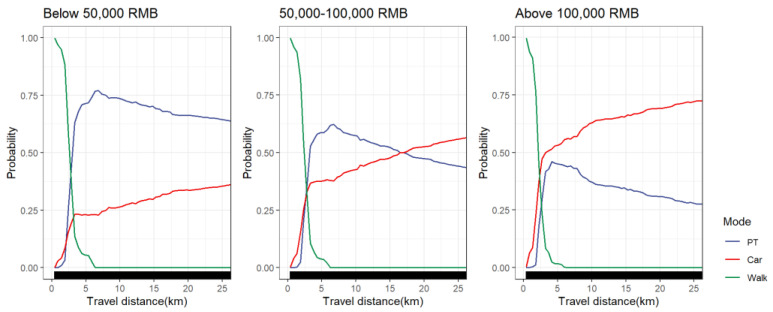
PDP plot of household income groups differences.

Differences in the nonlinear effect of travel distance on travel mode choice between household income groups are shown in Figure 13. It can be seen that the probability of choosing cars showed an upward trend among the three groups in the range of 0–3 km. At 3 km, the choice probability of cars was 24% for travelers with low-income households (below RMB 50,000), 37% for travelers with middle-income households (between RMB 50,000 and 100,000) and 50% for travelers with high-income households (above RMB 100,000). The probability of choosing cars slowly increased after the travel distance exceeded 3 km. The choice probability of public transportation peaked at 4 km for travelers with high-income households, with the largest percentage below 50%. For travelers with low-income households and middle-income households, the choice probability of public transportation peaked at 6 km, with the proportion exceeding 75%. Overall, the choice probability of cars was more than public transportation after 2.5 km for travelers from high-income households, while travelers from low-income families were just the opposite. Travelers from middle-income households tended to choose public transportation within a travel distance of 3–17 km, while they tended to choose a car when the travel distance exceeded 17 km. This is because travelers from high-income households have higher requirements for travel costs and comfort, and cars not only provide their travel needs but are often a symbol of power [7]. However, for the low-income class, the probability of owning a car is relatively low, and the freedom of housing location selection is low. They usually need to pay more travel time and travel costs in exchange for the low cost of housing. Therefore, public transportation must become the main mode of medium and long-distance travel.

#### 4.5.7. Travel Purpose Groups

**Figure 14:**
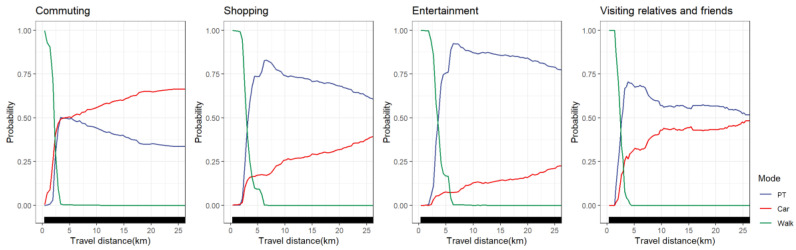
PDP plot of travel purpose groups differences.

Differences in the nonlinear effect of travel distance on travel mode choice between travel purpose groups are shown in Figure 14. For commuters, the probability of choosing to walk was nearly 0 when the travel distance was over 3 km. Those visiting relatives and friends no longer chose to walk after 4 km, while those traveling for shopping and entertainment accounted for approximately 50% at 3 km, with the probability of choosing to walk nearly 0 at around 6 km. We found that the probability of choosing public transportation for commuters peaked at approximately 3 km (accounting for roughly 50%). After the distance threshold of 3 km was exceeded, the probability of choosing public transportation for commuters began to decline gradually, while the probability of choosing cars slowly increased. The probability of choosing public transportation for the group visiting relatives and friends peaked at 4 km, accounting for 68%. The probability of choosing public transportation for the shopping and entertainment groups had a relatively high peak (larger than 85% at about 6 km). We found that among non-commuting groups, the probability of choosing cars was less than 50%, and especially for entertainment residents, the probability of choosing cars was less than 30%. Overall, commuters chose cars when the travel distance exceeded 3 km, while public transportation accounted for a large proportion of non-commuter travel after 3 km. This is because commuters have a relatively high demand for travel time, and cars have a higher advantage in long-distance travel.

## 5. Discussion and Conclusions

The relationship between travel distance and mode choice has been the focus of researchers, but there is still a lack of research on the nonlinear association and group heterogeneity between both of them. Based on survey data from Guiyang City, China, this paper explores the nonlinear association and group differences between travel distance and travel mode choice using a random forest model.

As expected, we found that travel distance is more important than built environment variables for understanding the mechanism of travel mode choice. Travel distance has the largest contribution of explanatory power for predicting travel mode choice, with a contribution of 42.28%, and even contributes 63.24% of explanation power for walking. The relative importance of car ownership ranks second in predicting travel mode choice, accounting for 10.43%. In addition, car ownership makes the largest contribution to predicting car choice at 29.22%, but its relative importance in predicting walking choice is only 3.44%. We also found that the relative importance of the built environment decreases significantly, from 58.96% to 24.80%, when the travel distance variable is taken into account in the model. This phenomenon confirms the importance of travel distance in the mode choice model, where travel distance may weaken the relative importance of the built environment. We call on researchers and planners to deeply explore the potential mechanism of travel behavior and make decisions with a cautious and sensible attitude when evaluating the importance and impact of the built environment [1].

This study confirms that travel distance has nonlinear and threshold effects on travel mode choice. In this study, the choice probability of walking is negatively correlated with travel distance. The probability of choose walking within 1.4 km decreases slowly. From 1.4 km to 3.5 km, the probability of walking choice decreases sharply in the form of an almost linear change of approximately 90 percent. The probability of choosing a car increases nonlinearly with travel distance, and there is a significant threshold effect between travel distance and public transportation mode choice. The probability of choosing public transportation peaks at approximately 6 km. Understanding how travel distance affects travel mode-choosing behavior can help policymakers optimize public transportation infrastructure and non-motorized transportation environments. This study shows that before 2.5 km, the choice probability of walking is greater than the car and public transportation. We recommend planners optimize pedestrian infrastructure within 2.5 km of residential areas, especially planning for pedestrian-friendly environmental communities within 1.4 km. Within 2.5–12.5 km of residential areas, planners should focus on optimizing the layout of public transportation infrastructure, such as increasing public transportation routes, reducing the number of transfers and developing customized buses and community buses to encourage residents to use public transportation as much as possible in medium and long-distance trips and reduce the use of cars by urban residents.

Further, the nonlinear effect of travel distance on travel mode choice was significantly different among different groups. The increase in travel distance may encourage travelers to give up walking and choose a faster travel mode, but we found that the increase in travel distance does not encourage all travelers to choose cars. There is also significant group heterogeneity in the choice behavior of travel patterns. In this paper, male travelers, travelers aged 31–45, commuters, people with regular jobs (including public institutions and civil servants and enterprise staff), people with higher education levels, travelers from high-income families and travelers with household car availability have a relatively lower probability of walking more than 3 km. When the travel distance exceeds 2.5 or 3 km, such travelers tend to choose cars. Most of these groups are relatively free in choosing their residence locations and show dependence on cars during their trips. As a result, they should be the target group guided to transfer to low-carbon transportation. A recent study indicates that travelers with higher affordability are more likely to frequently use customized buses (CB), and CB are more advantageous than public transportation for long-distance trips [44]. The CB system aims to provide high-quality bus transportation services for some specific types of travel demand. Planners should pay attention to planning the CB system for residents living in high-priced urban areas to reduce the frequent use of cars by such groups on long-distance trips. Policymakers can control and guide the ownership and use of family cars in such groups on a household basis, for example, by publishing policies to encourage ride-sharing and car-sharing in the residence to reduce the use of family cars. Another possible measure is to combine preferential public transit policies with reducing vehicle dependence. For example, car owners are encouraged to redeem their car license plates for free public transport passes. This policy has proven to be feasible in Flanders.

On the other hand, female travelers, the elderly (over 60 years old), unemployed or retired people, people with lower education levels, travelers from low-income families, non-commuter travel (shopping, leisure and entertainment, visiting relatives and friends) and travelers without household car availability have a relatively high probability of walking 0–2 km. When the travel distance exceeds 2.5 or 3 km, such travelers tend to choose public transportation. These groups usually have poor mobility. As Simcock et al. [59] stated, they are more likely to be limited by transport poverty. For such groups, the freedom of housing location selection is relatively low, and car ownership may be an economic burden. They usually cannot enjoy enough accessibility and social participation opportunities and thus face social exclusion. They are forced to use public transportation for medium and long-distance trips. Researchers, policymakers and planners should focus on groups that may be at risk of transportation poverty. In particular, it is necessary to ensure that they can obtain safe and comfortable public transportation services during trips and try to improve their dependence and satisfaction on public transportation. An effective measure is to use economic means to compensate for travel costs, such as discounted bus passes for the low-income class. In addition, government departments can provide subsidized policies such as affordable housing in areas with convenient transportation to help them improve the jobs–housing balance and increase the fairness of activity participation.

This study has some limitations. These nonlinear effects are likely to vary at spatial scales [60]. This study also cannot provide evidence for causality. In the future, we will establish the nonlinear difference of spatio-temporal relationships based on longitudinal data and explore the differences between different types of cities. Secondly, the attitudes and preferences for travel and the built environment are not considered because travelers’ choice of travel mode may also be caused by their self-choice. It is still necessary to consider more built environment data and attitude variables in the future, which can provide reliable evidence for the theory of the causal relationship between travel distance and mode choice. Thirdly, this study only focuses on the nonlinear influence of travel distance on the choice of travel mode. However, the relationship between travel mode choice and other travel information variables is still important. Thus, the nonlinear influence of other travel information, such as congestion time, on travel mode choice must be explored further in the future.

## Figures and Tables

**Table 1:** Descriptive statistics of explanatory variables.

Variables	Description	Mean	SD/Percentage
**Socio-demographics**			
Gender	Male = 1, female = 0.	0.47	0 = 47.0%, 1 = 53.0%
Age	18 to 30 years old = 1; 31 to 45 years old = 2; 46 to 60 years old = 3; older than 60 = 4.	2.38	1 = 18.5%, 2 = 37.8%, 3 = 31.0%, 4 = 12.7%
Education	Middle school and below = 1; high school to junior college = 2; undergraduate and above = 3.	2.09	1 = 30.4%, 2 = 30.3%, 3 = 39.4%
Occupation	Public institutions and civil servants = 1; enterprise staffs = 2; freelancers = 3; unemployed or retired = 4.	2.74	1 = 14.5%, 2 = 19.2%, 3 = 43.7%, 4 = 22.5%
Household size	Under three people = 1; three or more = 0.	0.29	0 = 29.0%, 1 = 71.0%
Household workers	Unemployed = 1; A single worker = 2; employed couple = 3; otherwise = 4.	2.65	1 = 11.1%, 2 = 23.8%, 3 = 54.0%, 4 = 11.1%
Household income	Below 50,000 RMB = 1; 50,000 RMB-100,000 = 2; above 100,000 = 3.	2.07	1 = 28.9%, 2 = 35.4%, 3 = 35.7%
Bicycle ownership	Owning one or more bicycles = 1; otherwise = 0.	0.11	0 = 88.9%, 1 = 11.1%
Car ownership	Owning one or more cars = 1; otherwise = 0.	0.61	0 = 39.3%, 1 = 60.7%
**Travel information**			
Travel purpose	Commuting = 1; shopping = 2; entertainment = 3; visiting relatives and friends = 4.	1.46	1 = 68.0%, 2 = 20.6%, 3 = 8.4%, 4 = 2.9%
Travel distance	Travel distance (km).	5.36	6.86
**Built environment**			
Land use mix	Mixture of residential, public management and services, commercial services, industry, parks and squares.	0.37	0.06
Road network density	The length of road centerline per square kilometer (km/ km^2^).	5.57	2.19
Population density	Total population/Buffer area (10^3^ people/ km^2^).	18.25	18.86
Distance to CBD	Distance from house to the city center (km).	8.98	6.12
Distance to nearest bus stop	Distance from house to the nearest bus stop (km).	0.20	0.20

**Table 2:** Comparison of model evaluation indexes.

Evaluation Indicators				
	Accuracy	Precision	Recall	F1 Score
**RF-Model1**	0.830	0.806	0.815	0.811
**RF-Model2**	0.695	0.669	0.679	0.674
**MNL-Model1**	0.795	0.759	0.782	0.770
**MNL-Model2**	0.619	0.577	0.603	0.589

**Table 3:** Relative importance of feature variables (RF-Model1/RF-Model2).

	RF-Model1			RF-Model2		
Variable	Importance (%)	Rank	Total (%)	Importance (%)	Rank	Total (%)
**Travel information**						
Travel distance	42.28	1	45.85	--	--	6.72
Travel purpose	3.57	9		6.72	7	
**Built environment**						
Distance to CBD	6.62	3	24.80	12.53	1	58.96
Population density	5.96	4		12.05	2	
Road network density	5.68	5		11.71	3	
Land use mix	5.65	6		11.69	4	
Distance to nearest bus stop	5.44	7		10.97	5	
**Socio-demographics**						
Gender	3.86	8	29.35	5.14	8	34.32
Occupation	2.68	10		4.61	9	
Age	2.03	11		3.58	10	
Education	1.43	14		2.76	13	
Car ownership	10.43	2		9.61	6	
Bicycle ownership	0.52	16		1.12	15	
Household workers	1.65	12		3.05	11	
Household income	1.47	13		2.84	12	
Household size	0.74	15		1.61	14	
**Total relative importance**			100.00			100.00

**Table 4:** Relative importance of variables among the three travel modes in RF-Model1.

	Car		PT		Walk	
Variable	Importance (%)	Ranking	Importance (%)	Ranking	Importance (%)	Ranking
**Travel information**						
Travel distance	30.52	1	43.84	1	63.24	1
Travel purpose	2.84	7	3.38	6	5.75	2
**Built environment**						
Distance to CBD	5.32	4	8.54	3	5.11	3
Population density	4.81	6	3.19	7	4.79	4
Road network density	2.40	8	3.10	8	2.84	7
Land use mix	1.49	12	2.88	9	2.12	10
Distance to nearest bus stop	1.81	10	1.98	11	1.68	11
**Socio-demographics**						
Gender	9.56	3	3.77	5	2.30	8
Occupation	5.30	5	3.79	4	3.51	5
Age	2.29	9	2.10	10	2.24	9
Education	1.70	11	0.80	14	0.85	13
Car ownership	29.22	2	18.86	2	3.44	6
Bicycle ownership	0.12	16	0.27	16	0.10	16
Household workers	1.12	14	1.51	13	1.06	12
Household income	1.28	13	1.63	12	0.73	14
Household size	0.22	15	0.36	15	0.25	15
**Total relative importance**	100.00		100.00		100.00	

## Data Availability

The data used to support the findings of this study are available from the corresponding author upon request.
